# Carcinoid tumor of lung and BRCA mutation: a case report

**DOI:** 10.1186/s13256-019-2052-5

**Published:** 2019-05-01

**Authors:** Mohammed Z. Shariff, Diana Curras-Martin, Natasha Campbell, Varsha Gupta, John D. Mikhail, Michael J. Levitt, Mohammad A. Hossain

**Affiliations:** Internal Medicine Residency Program, Department of Medicine, Jersey Shore University Medical Center, Hackensack Meridian Health, Neptune, NJ 07753 USA

**Keywords:** Carcinoid tumor, BRCA mutation, Infrahilar mass

## Abstract

**Background:**

A BRCA mutation is a mutation in either of the *BRCA1* or *BRCA2* genes, which are tumor suppressor genes. Hundreds of different types of mutations in these genes have been identified, some of which have been determined to be harmful, whereas others have no proven impact. BRCA mutations are well known to be associated with breast, uterine, and ovarian cancers along with some nongynecological malignancies involving the peritoneum, prostate, pancreas, skin, stomach, and rectum. However, there are no reported cases to date of an association between carcinoid tumors and a BRCA mutation.

**Case presentation:**

Our patient was a 33-year-old White woman with BRCA2 mutation who presented to her primary care physician for evaluation of abdominal pain. She underwent computed tomography of her abdomen and pelvis, which showed an incidental finding of infrahilar mass along with renal stones. Further workup with bronchoscopy and biopsy of the mass confirmed it to be a carcinoid tumor of the lung.

**Conclusions:**

No literature thus far exists describing a connection between BRCA mutations and carcinoid tumors. Early diagnosis and prompt treatment of carcinoid tumors are proven to have impact on survival and prognosis of these patients.

## Background

Carcinoid tumors, also known as neuroendocrine tumors (NETs), are an uncommon group of pulmonary neoplasms accounting for 1–2% of all lung cancers in adults and around 20–30% of all NETs [1–4]. These tumor cells are most probably derived from peptide- and amine-producing neuroendocrine cells. NETs can be seen throughout the body, but the most common site is the gastrointestinal tract, and the second most common site is the lung. Multiple previous studies showed association of carcinoid tumor with family history of cancer, body mass index, diabetes mellitus, cigarette smoking, and alcohol consumption [5–8]. However, to the best of our knowledge, there has been no study or case reported yet implicating possible association of carcinoid tumors with BRCA gene mutation. With this case report, our objective is to create awareness among primary care physicians that carcinoid tumors can occur in patients with BRCA gene mutations, because early diagnosis and prompt treatment of these tumors can have high impact on prognosis.

## Case presentation

**Fig. 1:**
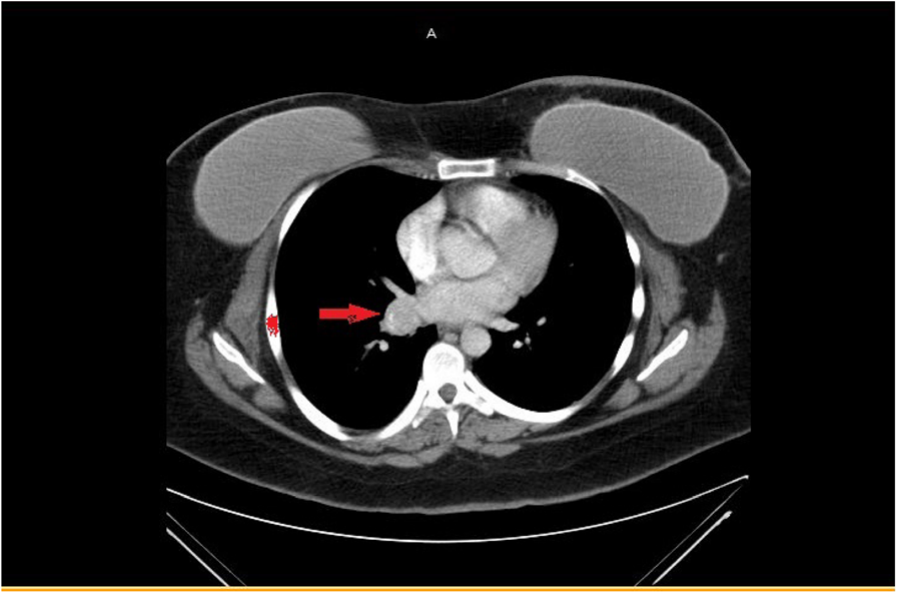
Computed tomographic scan of the chest showing a small lung nodule around the right hilum (*arrow*)

Our patient was a 33-year-old White woman with *BRCA2* gene mutation status. She presented to her primary care doctor for abdominal and back pain after bilateral prophylactic mastectomy. She had a strong family history of BRCA2-positive breast cancer in two of her aunts and one cousin. Her physical examination revealed that her vital signs were normal, and her abdominal examination was unremarkable except for mild nonspecific lower abdominal tenderness. For evaluation of lower abdominal pain and back pain, she underwent computed tomography (CT) of the abdomen and pelvis, which showed nonobstructing bilateral renal calculi and incidentally showed right infrahilar adenopathy. She underwent further CT of the chest with contrast enhancement, which revealed a right hilar mass measuring 3 × 2.2 cm and suspicious for malignancy (Fig. 1). Routine laboratory blood work was within normal limits and unremarkable, and possible infectious etiology was also ruled out (Table 1).

**Table 1:** Summary of laboratory results

	Result	Reference range
White blood cell count	9100/μl	4500–11 ,000/μl
Neutrophils	54.5%	50–70%
Lymphocytes	34.2%	25–43%
Monocytes	8%	0–9%
Eosinophils	2.3%	0–9%
Basophils	0.7%	0–2%
Hemoglobin	13.6 g/dl	12–16 g/dl
Hematocrit	41.3%	35–48%
Platelet count	374,000/μl	140,000–440,000/μl
Blood urea nitrogen	6 mg/dl	5–25 mg/dl
Serum creatinine	0.66 mg/dl	0.44–1.00 mg/dl
Serum sodium	136 mmol/L	136–145 mmol/L
Serum potassium	3.8 mmol/L	3.5–5.2 mmol/L

### Clinical findings

A pulmonologist was consulted for further evaluation of the infrahilar mass. On further inquiry, the patient reported occasional nonproductive cough with expiratory wheezing. She underwent electromagnetic navigational bronchoscopy for biopsy of her 2-cm mass in the right hilum. Biopsy confirmed a carcinoid tumor of the right lung. Atypical cells were positive for Cam5.2, thyroid transcription factor 1, synaptophysin, chromogranin, and CD56. The patient was then evaluated by a thoracic surgeon for possible surgical excision of the mass. She underwent right video-assisted thoracic surgery (VATS) with right thoracotomy, right middle and lower lobectomy, and lymph node resection.

### Diagnostic assessment

**Fig. 2:**
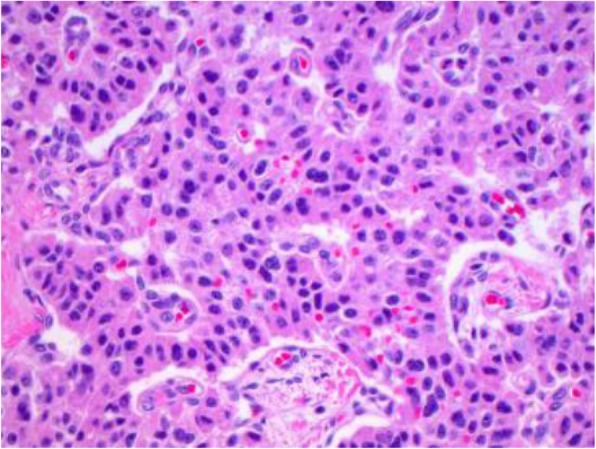
Histopathological slide of the right lung nodule demonstrating typical carcinoid tumor of the lung

The pathology report of the lung nodule confirmed it to be a typical carcinoid tumor with metastasis to one subcarinal lymph node (Fig. 2). A histopathological section of the lung nodule showed a neoplastic proliferation arranged in a nested and organoid pattern. The cells had monomorphic nuclei with “salt–and-pepper” chromatin and scant eosinophilic cytoplasm. Mitotic activity was low, and there was no necrosis. The patient’s tumor, node, metastasis staging is T2aN2M0.

### Therapeutic intervention

As per discussion with the patient and according to National Comprehensive Cancer Network (NCCN) guidelines, the patient would not receive any chemotherapy currently and would be closely followed by an oncologist.

### Follow-up and outcomes

The patient has had two follow-up appointments with her oncologist since the VATS procedure. After discussion with the patient and according to NCCN guidelines, the patient did not receive any adjuvant chemotherapy. The plan moving forward would be close surveillance with serial CT scans (3–6-month intervals) and chromogranin A measurements.

## Discussion

Carcinoid syndrome represents a constellation of symptoms that are seen in patients with NETs. The symptoms are exhibited because of release of chemical mediators, and as many as 40 such mediators have been identified in the pathophysiology of carcinoid syndrome [9]. The most prominent of these are serotonin, histamine, tachykinins, kallikrein, and prostaglandins. The most common presenting symptoms are cutaneous flushing (85%), diarrhea (80%), and bronchospasm leading to dyspnea and wheezing (20%) [10]. Less common presenting symptoms are venous telangiectasia and cardiac valve lesions. In our patient, these symptoms were identified during a retrospective systems review after CT scan findings and referral to a pulmonologist. One should have a high suspicion of carcinoid tumor when wheezing is the only complaint, because assuming it to be bronchial asthma and treating it with a β-agonist can lead to intense and prolonged vasodilation [11].

The most significant and important finding in our patient is the history of positive *BRCA2* gene mutation. Carcinoid tumors have been well studied from the early 19th century. There have been multiple case reports and studies about carcinoid tumors of the lung; however, to the best of our knowledge, there is no literature thus far describing a connection between BRCA mutations and carcinoid tumors. A BRCA mutation is a mutation in either of the BRCA1 or BRCA2 genes, which are tumor suppressor genes [12]. Hundreds of different types of mutations in these genes have been identified, some of which have been determined to be harmful, whereas others have no proven impact. BRCA mutations are well known to be associated with breast, uterine, and ovarian cancers along with some nongynecological malignancies involving the peritoneum, prostate, pancreas, skin, stomach, and rectum. The present case report highlights a possible association between carcinoid tumors and *BRCA2* gene mutation. Our patient had heterozygous germline *BRCA2* mutation c.4884_4885del. This observation can be incidental and unrelated; however, further studies and vigilant case reporting can solidify this association.

Carcinoid tumors are commonly seen in the gastrointestinal tract with frequent metastasis to liver. The first description of carcinoid tumors was in the late 18th century by Lubarsch, followed by Oberndofer in 1907 [13]. In the early 1950s, there were six reported cases of bronchial carcinoid. In 1960, Maurice Joseph and R. R. Taylor reported a case of a 62-year-old woman who initially presented in 1952 with a 3-month history of cough and a 19-kg weight loss. A chest x-ray was suggestive of a tumor involving the upper lobe of the lung in that patient. Bronchoscopy was performed, which did not show any definitive disease. She was advised to undergo thoracotomy, which she refused, and she sought discharge against medical advice. She was followed as an outpatient, and her clinical state did not alter until November 1958, when she complained of pain in the left chest and worsening dyspnea. She refused admission at that time. However, she was hospitalized 2 months later with complaints of severe dyspnea, intermittent diarrhea, intermittent flushing of the upper face and hemithorax, and episodes of wheezing. At this stage, diagnosis of functioning carcinoid was suggested, which was confirmed by elevated levels of 5-hydroxyindoleacetic acid in the urine. She was treated symptomatically with diuretics, nicotinic acid, digoxin, chlorpromazine, and mersaryl. The patient did not want to undergo any invasive procedures, so she was discharged to a nursing home at the request of her family, where she died a few months later. The postmortem report confirmed the tumor to be a carcinoid tumor of the left lung involving the entire lung with metastasis to regional lymph nodes. That was the first reported case of lung carcinoid, to the best of our knowledge. Compared with our patient, who received prompt VATS with excision of the tumor, the definitive treatment was not provided, because the patient refused invasive procedures, leading to death with distant metastasis.

The definitive treatment for lung carcinoid depends on the extent of disease, pulmonary reserve, and distant metastasis [14]. For patients with localized lung NETs, surgical resection is the preferred treatment modality, provided there is adequate pulmonary reserve. For patients whose condition does not allow complete surgical resection, options for control of tumor growth include radiotherapy, with or without concurrent chemotherapy, and palliative endobronchial resection of the tumor [15, 16]. Our patient had a small carcinoid tumor localized to the lung with a good pulmonary reserve; hence, she was promptly treated with complete resection of the tumor.

The role of adjuvant chemotherapy after complete resection of the lung NET is not recommended, owing to total lack of prospective randomized controlled trials. However, there are a number of retrospective studies to study the role of adjuvant chemotherapy, but most of those studies have not found a benefit from adjuvant chemotherapy [14–18]. Most of the patients included in the retrospective studies had a large NET of the lung that might have been responsible for the lack of benefit from the adjuvant chemotherapy. However, our patient has a small carcinoid tumor of the right lung with metastasis to only one subcarinal lymph node, so she might benefit from the adjuvant chemotherapy. However, as per the discussion with the patient, she did not receive any chemotherapy and would be closely monitored by her oncologist.

## Conclusions

No literature exists showing a connection between BRCA mutations and carcinoid tumors; thus, further studies and vigilant case reports are needed to solidify the association between them. Patients with BRCA mutation should be examined with a careful workup for carcinoid tumor in appropriate clinical scenarios. Delay in diagnosis may possibly result in unfavorable prognosis, which may ultimately lead to death. Early diagnosis and prompt treatment with complete surgical resection have been proven to prolong survival.

## Abbreviations

CT: Computed tomography
NCCN: National Comprehensive Cancer Network
NET: Neuroendocrine tumor
VATS: Video-assisted thoracic surgery
